# Supplementary material for: Co‐infection with Chikungunya virus alters trafficking of pathogenic CD8+ T cells into the brain and prevents Plasmodium‐induced neuropathology
Source: EMBO Mol Med. 2017 Nov 7;10(1):121–38. doi: 10.15252/emmm.201707885 (PMC5760855; doi:10.15252/emmm.201707885)
Supplement: Supplementary file 1 — Appendix [file EMMM-10-121-s001.pdf]

TABLE OF CONTENTS

APPENDIX FIGURES AND FIGURE LEGENDS

Appendix fig S1 -- 2

Appendix fig S2 -- 4

Appendix fig S3 -- 6

Appendix fig S4 -- 7

Appendix fig S5 -- 8

Appendix fig S6 -- 9

Appendix fig S7 -- 10

Appendix fig S8 -- 11

Appendix Figure S1

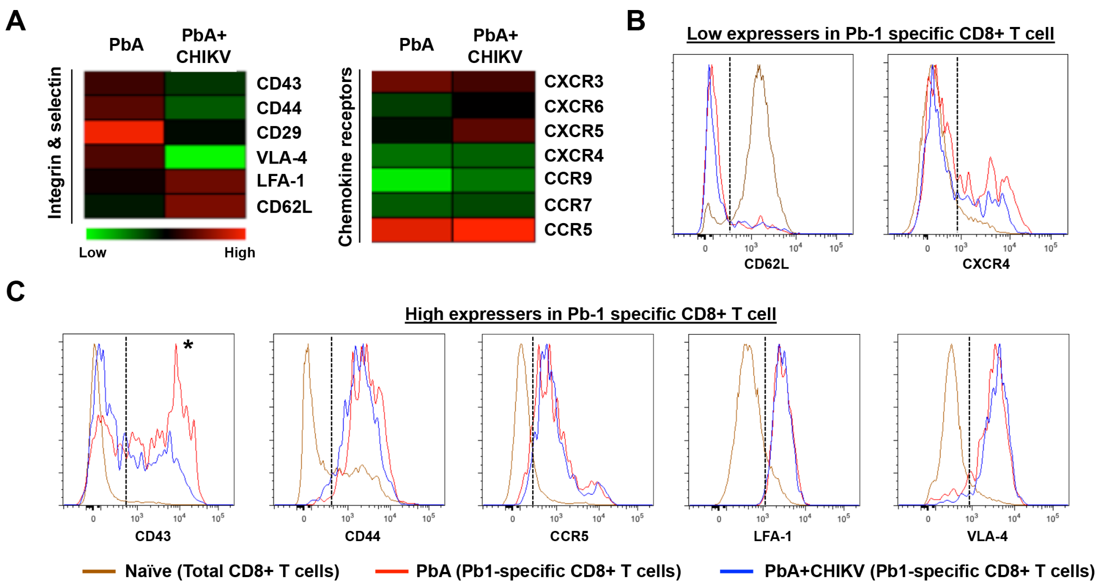

Appendix Figure S1. Regulation of chemotactic markers in CD8 T cells during concurrent co-infection on 6 dpi. (**A**) Gene expression of integrins, selectins or chemokine receptors that are differentially regulated in isolated CD8+ T cells from the spleen during concurrent co-infection on 6dpi. All data in the heat map are expressed as fold change relative to expression level in CD8+ T cells isolated from naïve mice. Gene expressions were done in duplicate using sorted CD8+ T cells pooled from naïve (n = 8), PbA (n = 15) and PbA+CHIKV (n = 6). Representative histograms showing surface expression of (**B**) low expressers (CD62L and CXCR4) and (**C**) high expressers (CD43, CD44, CCR5, LFA-1 and VLA-4) in Pb1-specific CD8+ T cells isolated on 6 dpi from PbA-luc or PbA-luc+CHIKV (n > 4 per group) mice. Expression levels of these markers in total CD8+ T cells of naïve mice (n > 4) were shown as a control because the numbers of Pb1-specific T cells in naive mice are below the level of detection in naïve mice using the tetramers. Threshold for positive staining of respective markers were represented by dotted line in each histogram. Markers with significant down-

regulation of surface expression (MFI) in the co-infected mice were indicated with *.

Appendix Figure S2

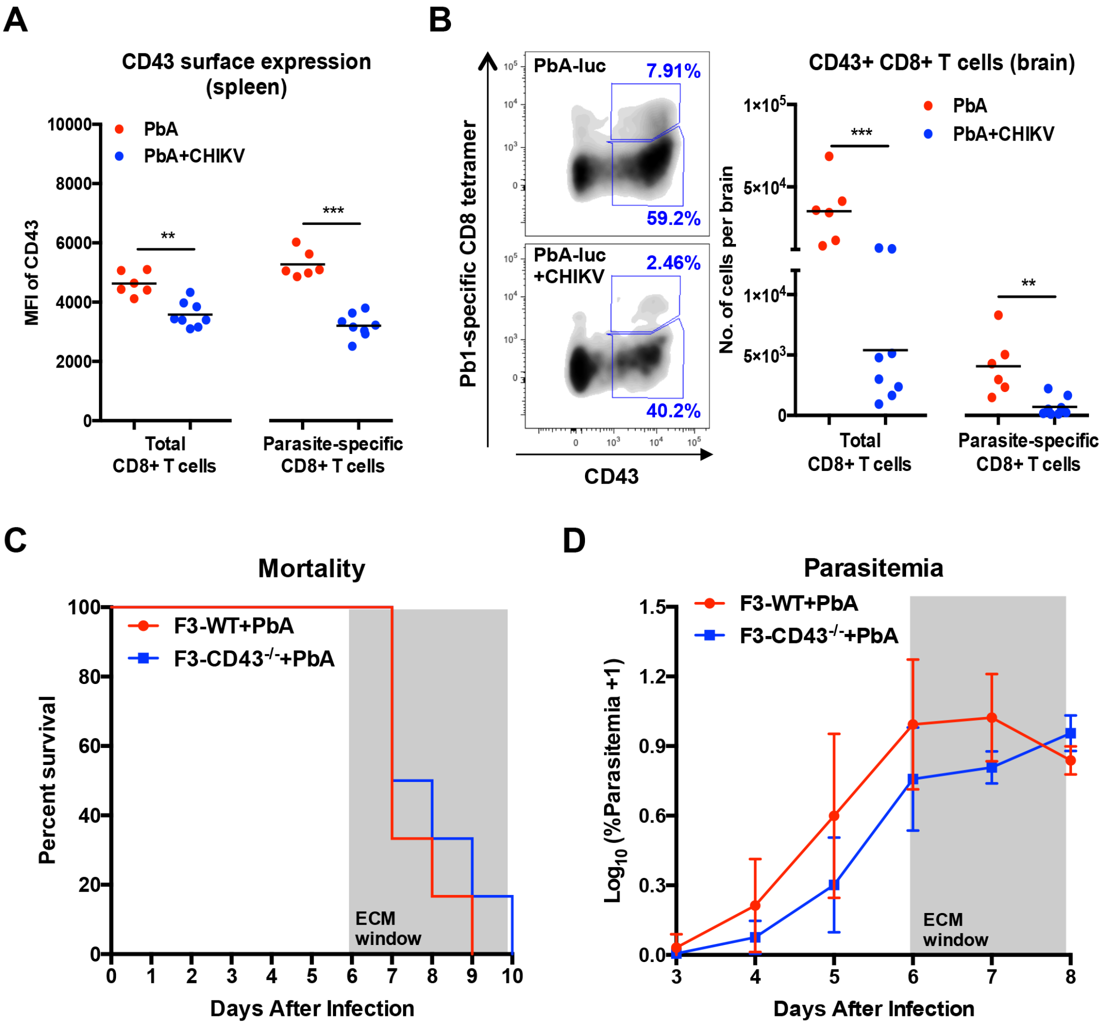

Appendix Figure S2. CD43 does not have a role in ECM induction. (**A**) Surface expression of CD43 in total CD8+ T cells and parasite-specific CD8+ T cells in the spleen of PbA (*n* = 6) and PbA+CHIKV (*n* = 8) on 6 dpi. (**B**) Numbers of CD43+ total and parasite-specific CD8+ T cells in the brain of PbA (*n* = 6) and PbA+CHIKV (*n* = 8) on 6 dpi. Representative flow-cytometry plots of CD8+ T cells profile in the brain showing reduced CD43+ total and parasite-specific CD8+ T cells upon co-infection on 6 dpi was shown. Data were analyzed by Mann-Whitney 2-tailed analysis (**P < 0.01 and ***P < 0.001). (**C**) Mortality curve and (**D**) parasitemia of PbA infected F3-WT (*n* = 6)

and F3-CD43$^{-/-}$ littermates (*n* = 6). F3 littermates were obtained by backcrossing CD43$^{-/-}$ in B6.129 background with C57BL/6J for 3 generations and deficiency of CD43 were determined by PCR genotyping.

Appendix Figure S3

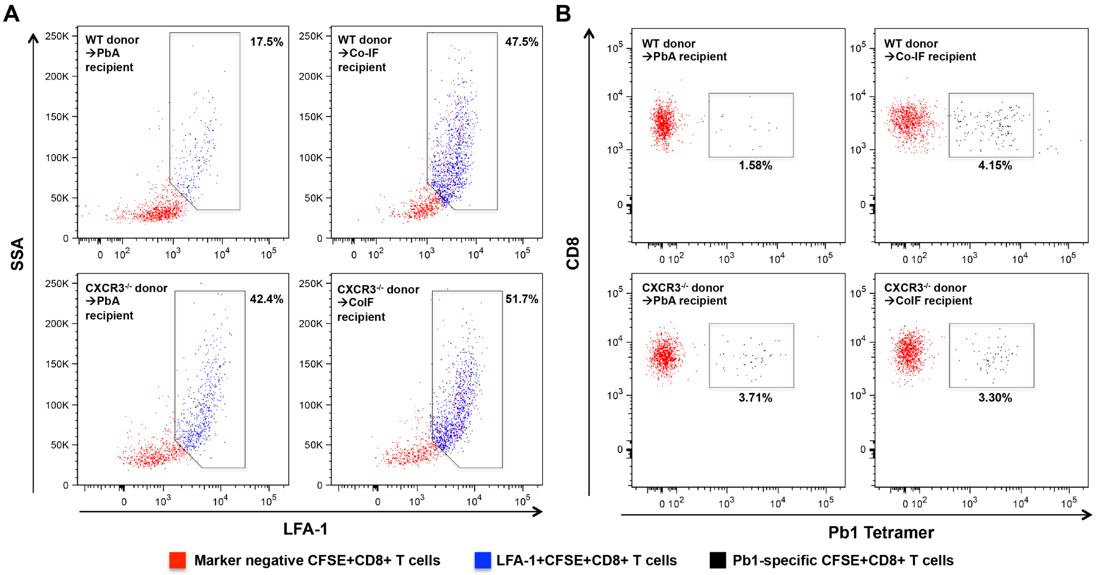

Appendix Figure S3. Reduction of activated and Pb1-specific CD8+ T cells retention in co-infected recipients with CXCR3$^{-/-}$ donors. Representative dot plots showing recovered (**A**) CFSE+LFA-1+ CD8+ T cells and (**B**) CFSE+Pb1-specific CD8+ T cells in the spleen of WT Donor→PbA recipient (*n* = 4), WT Donor→CoIF recipient (*n* = 5), CXCR3$^{-/-}$ Donor→PbA recipient (*n* = 4) and CXCR3$^{-/-}$ Donor→CoIF recipient (*n* = 5). Donor cells were labeled with CFSE. All dot-plots display CFSE+CD45+CD3+CD8+ T cells and were normalized to display the same number of events.

Appendix Figure S4

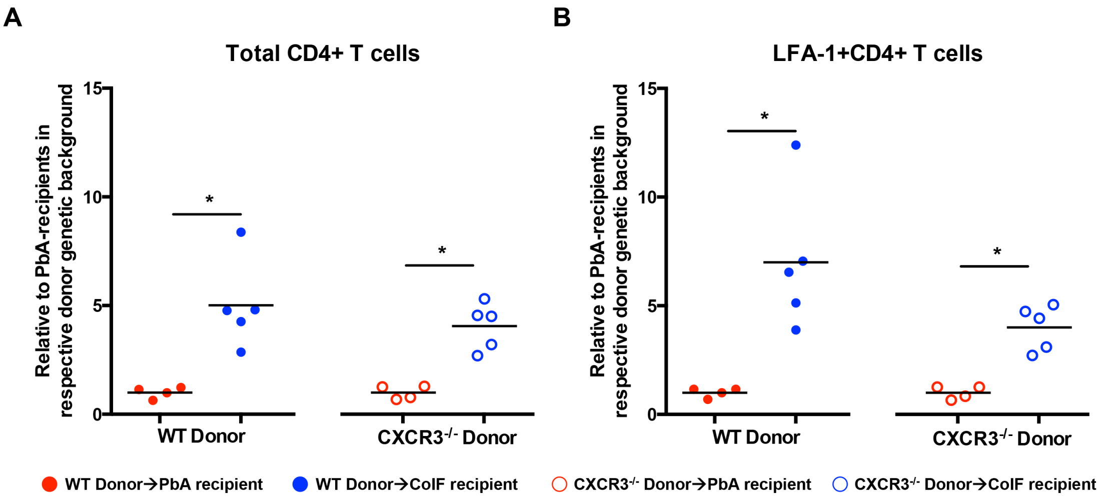

Appendix Figure S4. *In vivo* retention assay of CD4+ T cells. *In vivo* retention assay displaying fold increase of recovered donors' cells relative to the mean of recovered cells in PbA-recipients of their respective genetic backgrounds for (**A**) total and (**B**) LFA-1+ CD4+ T cells in each recipient spleen. WT Donor→PbA recipient (*n* = 4), WT Donor→Co-infected recipient (*n* = 5), CXCR3$^{-/-}$ donor→PbA recipient (*n* = 4) and CXCR3$^{-/-}$ donor→Co-infected recipient (*n* = 5). Data were analyzed by Mann-Whitney 2-tailed analysis (*$P < 0.05$). All recipients at 5 dpi received 5 x 10^7 splenocytes from pooled WT PbA-luc or CXCR3$^{-/-}$ infected donors (*n* = 10 per group; 6 dpi).

Appendix Figure S5

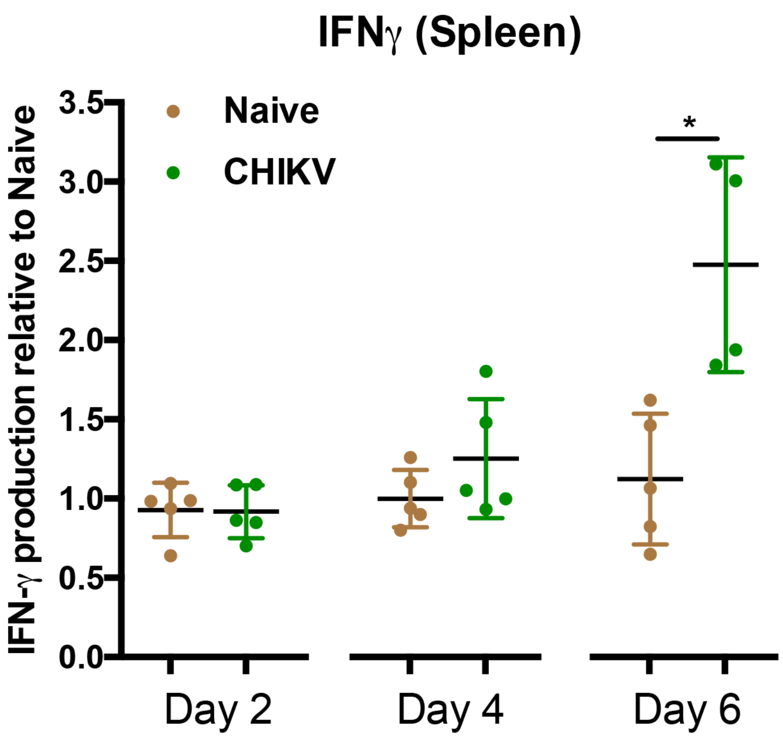

Appendix Figure S5. IFNγ induction profile in the spleen during CHIKV infection. IFNγ level in the cell lysate from the spleen of naïve and CHIKV-infected mice on 2 dpi, 4 dpi and 6 dpi ($n \geq 4$). IFNγ level in the cell lysate were determined using IFNγ ELISA and expression level was normalized relative to the respective naïve mice harvested in the same experiment. Each data point in the dot-plot represents data collected from 1 mouse.

Appendix Figure S6

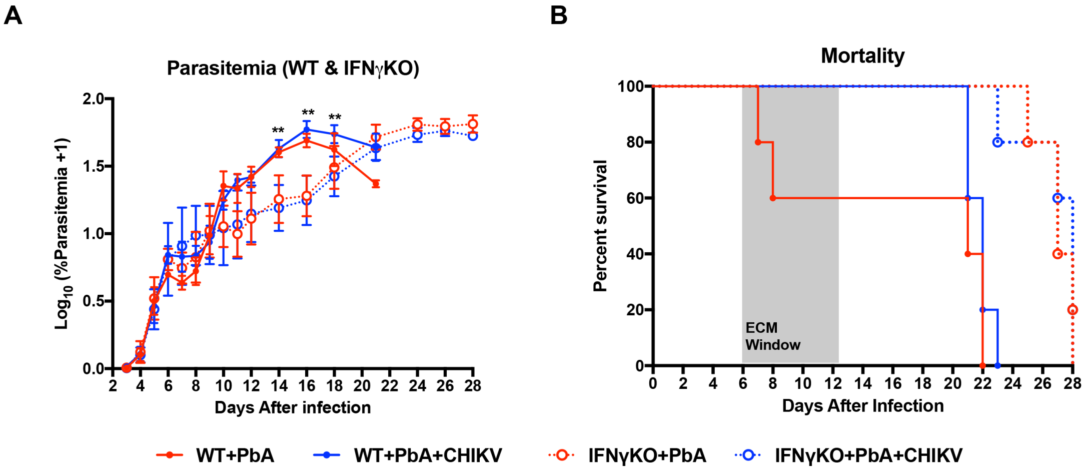

Appendix Figure S6. IFNγ$^{-/-}$ mice are protected ECM during single PbA or concurrent co-infection. (**A**) Parasitemia and (**B**) mortality of WT+PbA (*n* = 5), WT+PbA+CHIKV (*n* = 5), IFNγ$^{-/-}$+PbA (*n* = 5) and IFNγ$^{-/-}$+PbA+CHIKV (*n* = 5). Data of WT+PbA+CHIKV and IFNγ$^{-/-}$+PbA+CHIKV were compared by Mann-Whitney 2-tailed analysis (***P* < 0.01).

Appendix Figure S7

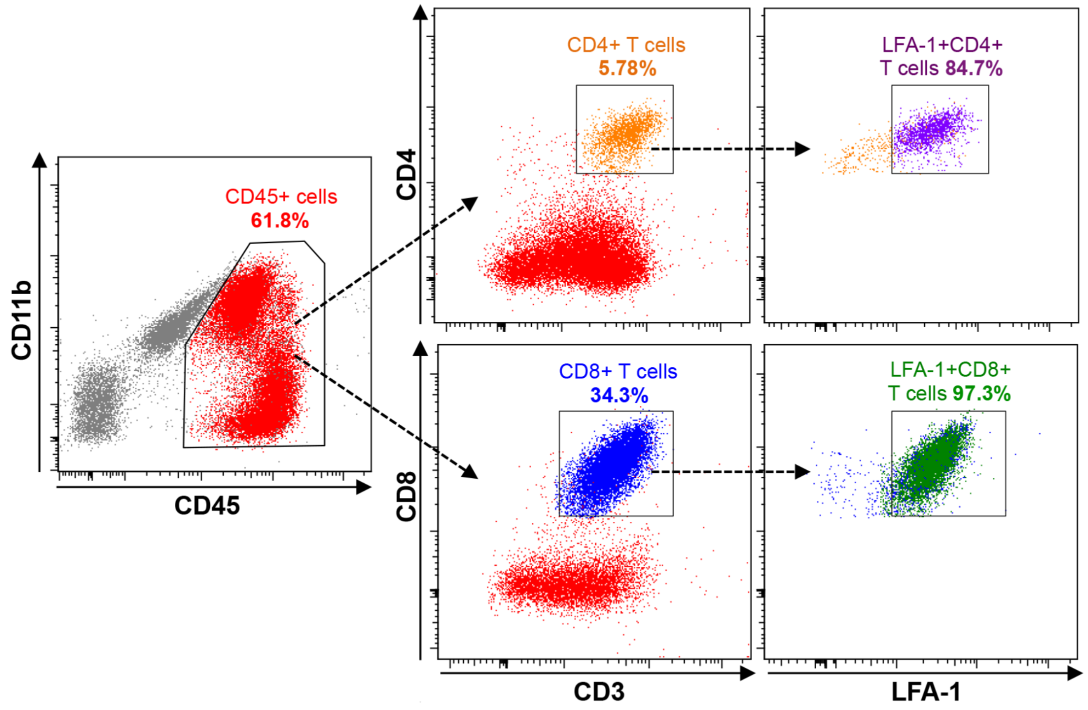

Appendix Figure S7. Representative gating strategy for T cell infiltrates in the brain on 6 dpi. Total and activated CD4+ T cells in the brain were defined by CD45hi/CD3+/CD4+ and CD45hi/CD3+/CD4+/LFA-1+ cells respectively. Total and activated CD8+ T cells in the brain were defined by CD45hi/CD3+/CD8+ and CD45hi/CD3+/CD8+/LFA-1+ cells respectively.

Appendix Figure S8

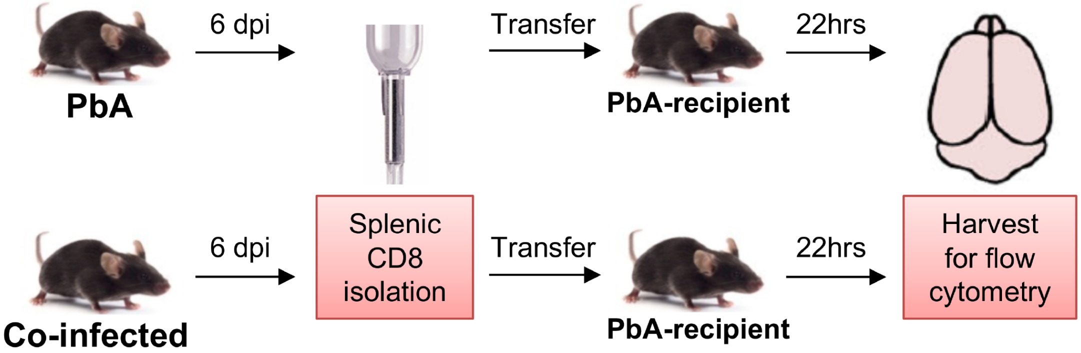

Appendix Figure S8. Schematic diagram of transfer protocol for *in vivo* migration assay. On 6 dpi, CD8+ T cells were isolated from the spleen of PbA donors or co-infected donors. Equal numbers of CD8+ T cells from each group of donors were transferred into PbA-infected recipients at 5 dpi. Recovery of CFSE-tagged transferred cells in the brain was profiled using flow cytometry.
